# Antimicrobial activity of bovine NK-lysin-derived peptides on bovine respiratory pathogen *Histophilus somni*

**DOI:** 10.1371/journal.pone.0183610

**Published:** 2017-08-21

**Authors:** Rohana P. Dassanayake, Shollie M. Falkenberg, Robert E. Briggs, Fred M. Tatum, Randy E. Sacco

**Affiliations:** Ruminant Diseases and Immunology Research Unit, National Animal Disease Center, United States Department of Agriculture, Agricultural Research Service, Ames, Iowa, United States of America; nanyang technological university, SINGAPORE

## Abstract

Bovine NK-lysins, which are functionally and structurally similar to human granulysin and porcine NK-lysin, are predominantly found in the granules of cytotoxic T-lymphocytes and NK-cells. Although antimicrobial activity of bovine NK-lysin has been assessed for several bacterial pathogens, not all the important bacterial pathogens that are involved in the bovine respiratory disease complex have been studied. Therefore the objective of the present study was to evaluate the antimicrobial activity of bovine NK-lysin-derived peptides on bovine respiratory pathogen *Histophilus somni*. Four, 30-mer peptides corresponding to the functional region of NK-lysin helices 2 and 3 were synthesized and assessed for antibacterial activity on four bovine pneumonic *H*. *somni* isolates. Although there were some differences in the efficiency of bactericidal activity among the NK-lysin peptides at lower concentrations (2–5 μM), all four peptides effectively killed most *H*. *somni* isolates at higher concentrations (10–30 μM) as determined by a bacterial killing assay. Confocal microscopic and flow cytometric analysis of Live/Dead *Bac*light stained *H*. *somni* (which were preincubated with NK-lysin peptides) were consistent with the killing assay findings and suggest NK-lysin peptides are bactericidal for *H*. *somni*. Among the four peptides, NK2A-derived peptide consistently showed the highest antimicrobial activity against all four *H*. *somni* isolates. Electron microscopic examination of *H*. *somni* following incubation with NK-lysin revealed extensive cell membrane damage, protrusions of outer membranes, and cytoplasmic content leakage. Taken together, the findings from this study clearly demonstrate the antimicrobial activity of all four bovine NK-lysin-derived peptides against bovine *H*. *somni* isolates.

## Introduction

Antimicrobial proteins were first described in early 1960s with the identification of three basic proteins with bactericidal activities from a lysosomal fraction of guinea-pig polymorphonuclear leukocytes [1]. Based on the molecular masses and antimicrobial activities, these proteins were later named as antimicrobial peptides (AMPs). The AMPs, or host defense peptides, are a diverse group of molecules which play an important role in the innate immune response and have been isolated from bacteria, insects and other invertebrates, birds, fish, amphibians, plants, and mammals [2–5]. Based on the amino acid composition and structure, AMPs are divided into several subgroups such as anionic peptides, linear cationic α-helical peptides, cationic peptides enriched for specific amino acids, anionic and cationic peptides that contain cysteine and form disulfide bonds, and anionic and cationic peptide fragments of larger proteins [6]. The majority of AMPs are known to interact with the lipid bilayer, leading to formation of ion channels, transmembrane pores, and membrane rupture resulting in microbial cell lysis, although AMPs can also interact with other microbial intracellular targets [6].

Human granulysin as well as porcine and bovine NK-lysin, have also been described as AMPs [7–9]. Granulysin and NK-lysin are found in the cytosolic granules of cytotoxic T-lymphocytes and NK cells and have been found to exhibit activity against a variety of Gram positive and Gram negative bacteria, viruses, fungi, parasites and tumor cells [8–12]. Both granulysin and NK-lysin are structurally related to the saposin-like protein (SAPLIP) family of lipid binding proteins [8, 12, 13]. Although most mammalian species, including humans and swine, have only a single antimicrobial protein gene (granulysin or NK-lysin), the cattle genome is known to contain four functional NK-lysin genes, *NK1*, *NK2A*, *NK2B* and *NK2C* [10]. Gene expression analysis has revealed that while *NK1* and *NK2A* were highly expressed in the intestinal Peyer’s patches, while *NK2C* was highly expressed in the lungs [10, 14]. It has also been previously reported that the potent antimicrobial activity of bovine NK-lysin is associated with helix 2 through helix 3 regions [9, 10, 14].

Bovine respiratory disease complex (BRDC), also known as shipping fever, is the most common infectious disease affecting older weanling, stocker, or feeder calves causing extensive economic losses to the North American beef and dairy cattle industry [15, 16]. BRDC develop as a complex interaction between environmental stress factors, host factors, and multiple viral and bacterial infectious agents [17]. Various viral pathogens such as bovine herpes virus-1, bovine viral diarrhea virus, bovine respiratory syncytial virus, bovine parainfluenza-3, bovine coronavirus and bovine adenoviruses have been implicated to predispose calves to secondary bacterial infections. The most important bacterial pathogens associated with BRDC are *Mannheimia haemolytica*, *Pasteurella multocida*, *Histophilus somni*, and *Mycoplasma bovis* [15, 17]. Each of these bacteria are commensals in the nasopharynx of cattle. *H*. *somni* is a Gram negative bacterium and is a commensal of the upper respiratory as well as genitourinary tracts of cattle [15]. *H*. *somni* can cause a wide variety of disorders in cattle in addition to respiratory disease, including septicemia, thrombotic meningoencephalitis, arthritis, myocarditis, and abortions [18].

Antimicrobial activity of bovine NK-lysin derived peptides against BRDC pathogens *Mannheimia haemolytica*, and *Pasteurella multocida* has been reported [14]. It has also been previously reported that compared to *M*. *haemolytica* isolates, *P*. *multocida* isolates were less susceptible to bovine NK-lysin mediated killing. *M*. *haemolytica* isolates were susceptible for NK2A and NK2C, but they were resistant to NK1 and NK2B. However, *P*. *multocida* isolates were susceptibility to NK1 and NK2A, they were resistant to NK2B and NK2C. Since these two closely related pathogens showed differential sensitivity to bovine NK-lysins, it was of our interest to assess the sensitivity of the other most important BRDC associated pathogen *H*. *somni* to bovine NK-lysins. Therefore, all four bovine NK-lysin derived peptides corresponding to the functional region of helices 2 and 3 were synthesized and their antimicrobial activity on several bovine pneumonic isolates of *H*. *somni* were examined. Ultrastructural changes to the cell membrane of *H*. *somni* following incubation with NK2A peptide were also examined.

## Materials and methods

### NK-lysin peptide synthesis

As previously described [9, 10], four 30 amino acids long linear peptides corresponding to the functional regions of bovine NK-lysin (helices 2 and 3) of NK1 [VIIHVTSKVCSKMGLWSILCNQMMKKYLNR], NK2A [TVIEVASKMCSKMRLLKGLCKSITKRFLRR], NK2B [TVIEAASKVCGKMGPLKGLCKSITKRFLRR] and NK2C [TVIEEASKVCSKMRLLKGLCKSIMKKFLRT]) were synthesized by Peptide 2.0 Inc (Chantilly, VA), with over 95% purity. Lyophilized peptides (1 mg aliquot) were stored at -20°C, dissolved in phosphate-buffered saline (PBS, pH 7.4) and stored in aliquots again at -20°C until used.

### Bacterial isolates and culture conditions

Bovine pathogenic *Histophilus somni* pneumonia isolate (2336) was kindly provided by Dr. Lynette B. Corbeil at the Department of Pathology, School of Medicine, San Diego, CA [19] and three *H*. *somni* isolates (21, 22, and 91) which were originally isolated from pneumonic cattle lungs were kindly provided by the Veterinary Diagnostic Laboratory, College of Veterinary Medicine, Iowa State University. *Histophilus somni* isolates were maintained as frozen stocks (-80°C) in Columbia broth with 10% glycerol. All *H*. *somni* isolates were grown individually in trypticase soy agar supplemented with 5% defibrinated sheep blood (TSA II™, Becton, and Dickinson Co., Sparks, MD) at 37°C in a humidified atmosphere of 7.5% CO_2_ for 16 to 48 hrs.

### Antimicrobial killing assay

Antimicrobial killing assay was performed as described previously, but with minor modifications [9, 14]. Briefly, *H*. *somni* isolates grown on TSA sheep blood agar plates were collected using a cotton swab and diluted in Columbia broth to an optical density at 600 nm of 0.8 (~1 × 10^9^ colony forming units per milliliter (CFUs/ml)). The bacterial suspension was further diluted in PBS to obtain ~1–5 × 10^6^ CFU/ml. One hundred microliter aliquots of bacterial suspension (~1–5 × 10^5^ CFUs) were placed in a non-tissue culture treated flat-bottom 96-well plate (Becton Dickinson) and gently mixed with 20 μl of each of the diluted NK-lysin peptides (2, 5, 10, 20, and 30 μM final concentration) or PBS. The plate was covered with a lid and incubated at 37°C in a humidified atmosphere of 7.5% CO_2_ with constant shaking (~50 rpm) for 60 min. For quantitative counting, bacterial samples were then serially diluted in PBS, a 100 μl aliquot of each dilution was spread on TSA sheep blood agar plates and incubated at 37°C for up to 2 days. Bacterial colonies were enumerated for each dilution. Antimicrobial killing assays were repeated at least twice with three replicates for each peptide.

### Bacterial viability staining

To easily and reliably distinguish dead bacteria from live bacteria following incubation with NK-lysin peptides, *H*. *somni* was stained using LIVE/DEAD *Bac*Light bacterial viability kit as described by the manufacturer, but with some modifications (Cat no. L13152; ThermoFisher Scientific, Carlsbad, CA). Briefly, ~1 × 10^8^ cfu of *H*. *somni* suspension in 100 μl PBS was placed in a 96-well plate and 20 μM final concentration of each of the NK-lysin peptide was added and samples were incubated at 37°C in a humidified atmosphere of 7.5% CO_2_ with constant shaking for 30 min. Untreated and ethanol killed *H*. *somni* were used as live and dead controls, respectively. Fifty microliters of Syto 9 and 50 μl of propidium iodide (approximate final concentrations of Syto 9 and propidium iodide were 6 μM and 30 μM, respectively) were added to each well and incubated at 37°C for additional 15 min. The viability of *H*. *somni* was then determined by confocal laser-scanning microscopy and flow cytometry (green (live) versus red (dead) bacteria).

### Confocal laser-scanning microscopy

Approximately 150 μl of bacterial suspensions, previously incubated with 20 μM NK-lysin peptides or PBS along with Syto 9 and propidium iodide, were adhered on to glass slides using Shandon cytospin 2 and coversliped using *Bac*Light mounting medium (ThermoFisher). Live and dead bacteria were then visualized using Nikon A1R+ Confocal System microscope (Nikon Instruments, Melville, NY). Syto 9 and propidium iodide were excited at 488 nm and 561 nm solid state laser beam and emission signals 500–550 nm (green) 570–620 nm (red) were recorded, respectively. Images were collected using proprietary NIS-Elements Advanced Research software. Calibration was created for both dyes using the software and sequentially collected frames of individual channels were merged and saved as TIFF files. Images were obtained with plan Apo 60× objective lens (oiled) at numerical aperture 1.4. Final figures were prepared using Adobe Photoshop Elements 11.

### Flow cytometry

Two-color flow cytometric analyses was performed using a BD LSRII flow cytometer (BD Biosciences). *Histophilus somni* were visualized in forward and side light scatter and electronic gates were set to contain single bacterial cells. Single (Syto 9 or propidium iodide) and double fluorescence dye labeled live and dead bacteria were included to further optimize acquisition gates and compensation for each fluorochrome. Both Syto 9 and propidium iodide were excited at 488 nm laser beam and the emission signals were detected using a 530/30 nm and 575/25 nm long-pass filters, respectively. Approximately, 10,000 events were collected for data analysis and relative live/dead bacterial changes were determined using FlowJo software (FlowJo LLC, Ashland, OR).

### NK-lysin bacterial binding assay

To determine the binding of NK-lysin peptides with bacteria, *H*. *somni* was incubated with Cyanine dye 5 (Cy5)-conjugated NK2A and analyzed by flow cytometry and confocal microscopy. Since NK2A showed the highest antimicrobial activity, NK2A peptide was chosen for Cy5 labeling. Peptide-dye conjugation was performed with Lightning-Link^®^ Cy5 rapid conjugation system as described by the manufacturer (Innova BioSciences, Cambridge, UK). Approximately, 1 × 10^8^ CFU of *H*. *somni* suspension in 100 μl PBS was placed in a 96-well plate and incubated with either 20 μM final concentration of NK2A-Cy5 conjugate or similar amount of unconjugated Cy5 dye for 30 min at 37°C in a humidified atmosphere of 7.5% CO_2_ with constant shaking. Samples were then directly assessed by flow cytometry and confocal microscopy as describer earlier, but with Cy5 excitation (633 nm) and emission (660/20 nm) filters. In order to visualize NK-lysin binding to *H*. *somni* by confocal microscopy, coverslips were mounted using ProLong^®^ Gold antifade mount medium with DAPI (4`, 6`-diamidino-2-phenylindole).

### Propidium iodide uptake assay

To determine kinetics of NK-lysin induced damage to *H*. *somni* cell membrane, propidium iodide (PI) uptake assay was performed [20]. Briefly, ~1 × 10^8^ cfu of *H*. *somni* suspension in 100 μl PBS was placed in a 96-well opaque plate and mixed with 5 μl of PI (final concentration 3 μM). Samples were then incubated at 37°C in a humidified atmosphere of 7.5% CO_2_ with constant shaking for 5 min. After pre-staining *H*. *somni* with PI, different concentrations of NK2A peptide (1, 2, 5, and 20 μM final concentrations), 1% (v/v) Triton X-100, or kanamycin (50 μg/ml) were added into each well. At selected time points, fluorescent intensity of each sample was measured using a fluorescent microplate reader with excitation and emission at 530 nm and 620 nm, respectively. Ethanol-killed and Trion X 100 treated *H*. *somni* were used as positive controls and untreated and kanamycin treated *H*. *somni* were used as negative controls.

### Transmission electron microscopy

Approximately 2 × 10^9^ CFUs of *H*. *somni* in 100 μl of PBS was transferred into a 96-well plate and incubated with 30 μM of NK2A peptide or PBS at 37°C in a humidified atmosphere of 7.5% CO_2_ with constant shaking for 60 min. The bacterial suspension was mixed with an equal volume of 3% glutaraldehyde in 0.1 M cacodylate buffer (pH 7.4) and processed for electron microscopy essentially as described previously [10]. Briefly, after fixation, bacterial pellets were rinsed in cacodylate buffer, postfixed in 1% osmium tetroxide, dehydrated in alcohols, and embedded in epoxy resin. Ultrathin sections of the bacterial pellets were cut and stained with uranyl acetate and lead citrate. Sections were examined with a FEI Tecnai G2 Biotwin (FEI Co., Hillboro, OR) transmission electron microscope and images were captured with Advanced Microscopy Technologies (AMT Inc., Danvers, MN) imaging camera.

### Statistical analysis

Mean number of viable bacteria (CFUs/ml) and standard errors of means were determined. Student’s *t*-test was used to compare percentages of live and dead *H*. *somni* in control and NK-lysin treated samples. The term significant indicates *P* value of less than 0.05.

## Results

### Antimicrobial activity of bovine NK-lysin peptides on *H*. *somni*

The antimicrobial activity of bovine NK-lysin peptides against both Gram-positive, as well as Gram-negative bacteria, have been previously reported [9, 10]. Since *H*. *somni* is one of the primary bacterial pathogens involved in BRDC [15], a study was conducted to evaluate whether bovine NK-lysin peptides show antimicrobial activity against this bacterium, similar to that which has been reported earlier for two other BRDC causing bacterial pathogens, *M*. *haemolytica* and *P*. *multocida* [14]. To compare relative antibacterial activity of bovine NK-lysins on *H*. *somni*, we synthesized previously reported 30-mer peptides corresponding to the functional region of bovine NK-lysin helices 2 and 3 of NK1, NK2A, NK2B and NK2C [9, 10]. The antimicrobial activity of the peptides on *H*. *somni* were assessed using multiple techniques, including an antimicrobial killing assay, a Live/Dead *Bac*light bacterial viability assay with confocal microscopy and flow cytometry, as well as transmission electron microscopy.

**Fig 1:**
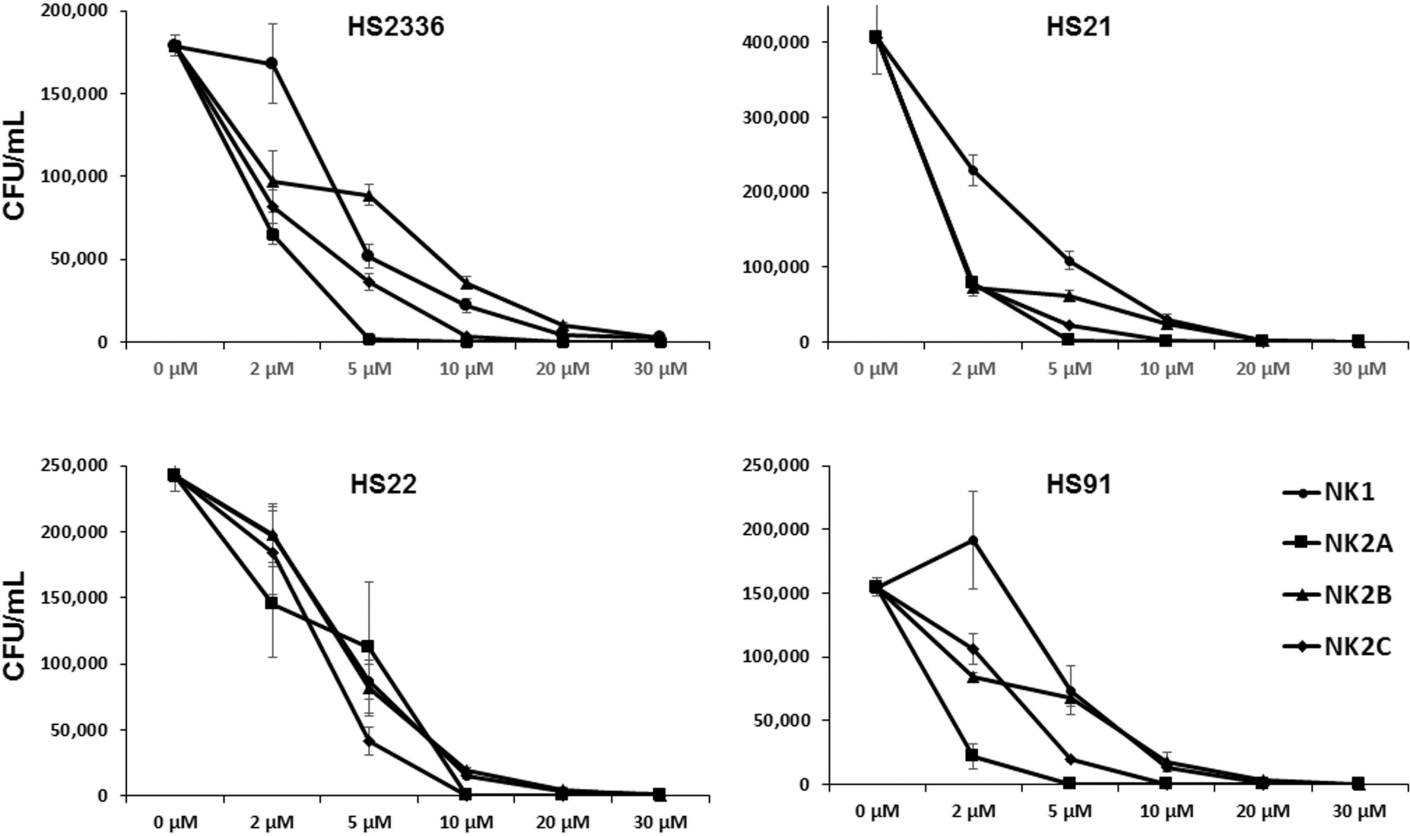
Antimicrobial activity of bovine NK-lysin-derived peptides on *H*. *somni* isolates. Live bacterial numbers (CFUs/ml) are shown after the incubation of indicated concentrations of NK-lysin peptides at 37°C for 60 min. Means and standard errors of means were calculated from two independent experiments with three technical replicates.

Bovine NK-lysin peptides killed *H*. *somni* isolates in a dose-dependent manner, with up to 100% of the bacteria killed during a 60 min incubation period (Fig 1). At higher concentrations (10–30 μM), all four peptides showed higher antibacterial activity against all four bovine *H*. *somni* isolates. However at lower peptide concentrations (2 and 5 μM), reduced antibacterial activity as well as variations in sensitivity among *H*. *somni* isolates was observed (Fig 1). Control *H*. *somni* isolates which were not incubated with any of the peptides but with PBS did not show detectable changes in the bacterial numbers (CFUs/ml) during 60 min incubation period (data not shown).

### Bovine NK-lysin peptides cause *H*. *somni* cell membrane damage

**Fig 2:**
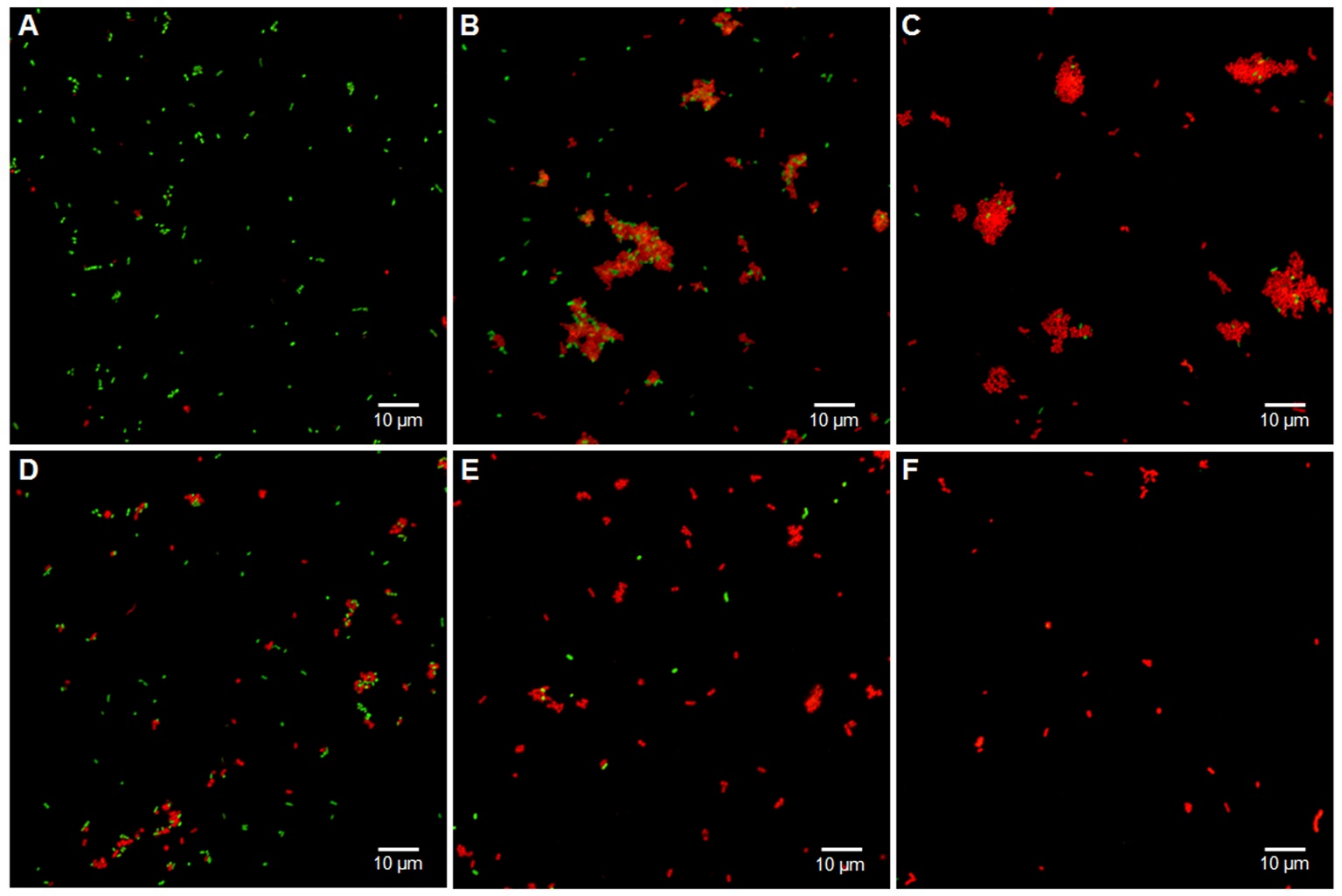
Confocal laser-scanning microscopic analysis of the viability of *H*. *somni* following incubation with bovine NK-lysin-derived peptides. Live and dead *H*. *somni* in control (A), NK1 (B), NK2A (C), NK2B (C), NK2C (E), and ethanol treated (F) samples. *H*. *somni* were incubated with 20 μM at 37°C for 30 min, and then stained with Live/Dead *Bac*light bacterial viability kit. Live bacteria with intact membranes (green) and dead bacteria with damaged membranes (red) were stained with Syto 9 and propidium iodide, respectively.

**Fig 3:**
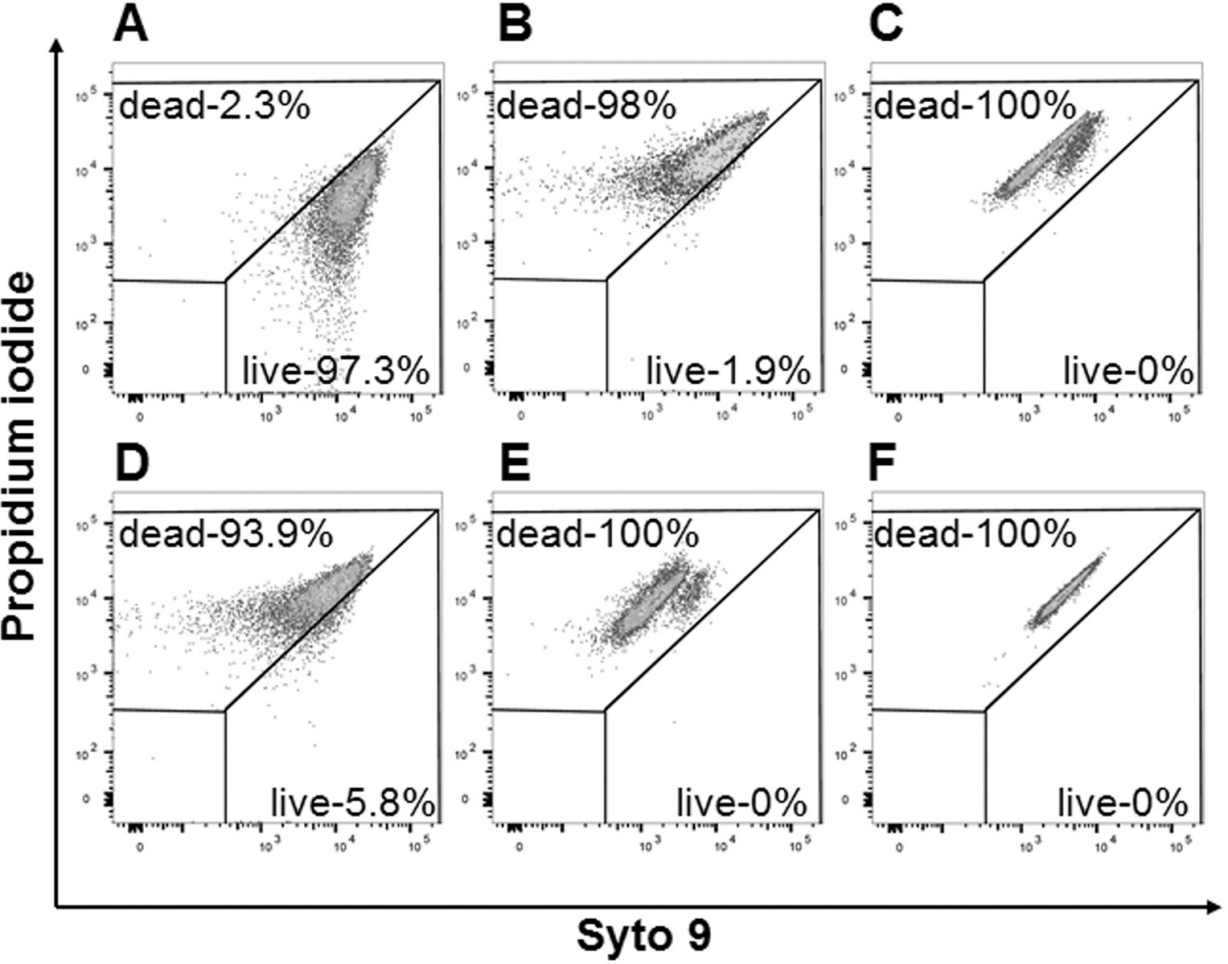
Flow cytometric analysis of the viability of *H*. *somni* following incubation with bovine NK-lysin-derived peptides. Live and dead *H*. *somni* in control (A), NK1 (B), NK2A (C), NK2B (D), NK2C (E), and ethanol treated (F) samples. *H*. *somni* were incubated with 20 μM at 37°C for 30 min, and then stained with Live/Dead *Bac*light bacterial viability kit. X axis indicates live bacteria (Syto 9) and Y axis indicates dead bacteria (propidium iodide).

Although the precise mechanism of antimicrobial activity of antimicrobial peptides (AMPs) is yet to be fully understood, previous studies report that AMPs can damage outer and inner bacterial membranes [6, 21]. *H*. *somni* isolates were sensitive to NK-lysin-derived peptides as evidenced by reduction in bacterial numbers (CFUs/ml) in killing assays (Fig 1) however, it was not clear whether the antibacterial activity of NK-lysin peptides on *H*. *somni* were bactericidal or bacteriostatic. Therefore to identify the mechanism of action, we performed Live/Dead bacterial viability staining after the incubation of *H*. *somni* with peptides. Syto 9 is a cell permeant green-fluorescent dye which can efficiently bind with both prokaryotic and eukaryotic DNA. Unlike Syto 9, propidium iodide is a cell non-permeant red-fluorescent dye and only stain DNA in the cells with compromised cell membranes. Therefore, we used Live/Dead bacterial viability staining kit containing both Syto 9 and propidium iodide to differentiate live from dead bacteria after incubation with NK-lysin peptides. As expected, majority of bacteria in control sample stained with Syto 9 (green, Fig 2A) and in killed bacteria stained with propidium iodide (red, Fig 2F). These findings confirmed that the cell membranes of live bacteria were intact. The damage to *H*. *somni* cell membranes was clearly visible as bacteria treated with NK-lysin peptides stained mostly with propidium iodide (Fig 2). Unlike with NK2B (Fig 2D) and NK2C (Fig 2E), clumping of dead *H*. *somni* were observed following incubation with NK1 (Fig 2B) and NK2A (Fig 2C). Concurrently, similarly incubated *H*. *somni* samples were also analyzed by flow cytometry. Since dead bacteria tended to clump, samples were thoroughly mixed before flow cytometry and electronic gates were positioned for single cell discrimination. Again as expected, most of the bacteria in the control sample were viable with intact cell membranes (Fig 3A) and all the bacteria treated with ethanol were found dead with compromised cell membranes (Fig 3F). Over 93% of *H*. *somni*, when incubated with NK-lysin peptides, were positive for propidium iodide staining or in the dead bacterial region further confirming the damage to the bacterial cell membranes (Fig 3B–3E). Taken together, both confocal microscopic and flow cytometric analysis suggest that all four NK-lysin peptides caused damage to *H*. *somni* cell membranes and are bactericidal in nature.

**Fig 4:**
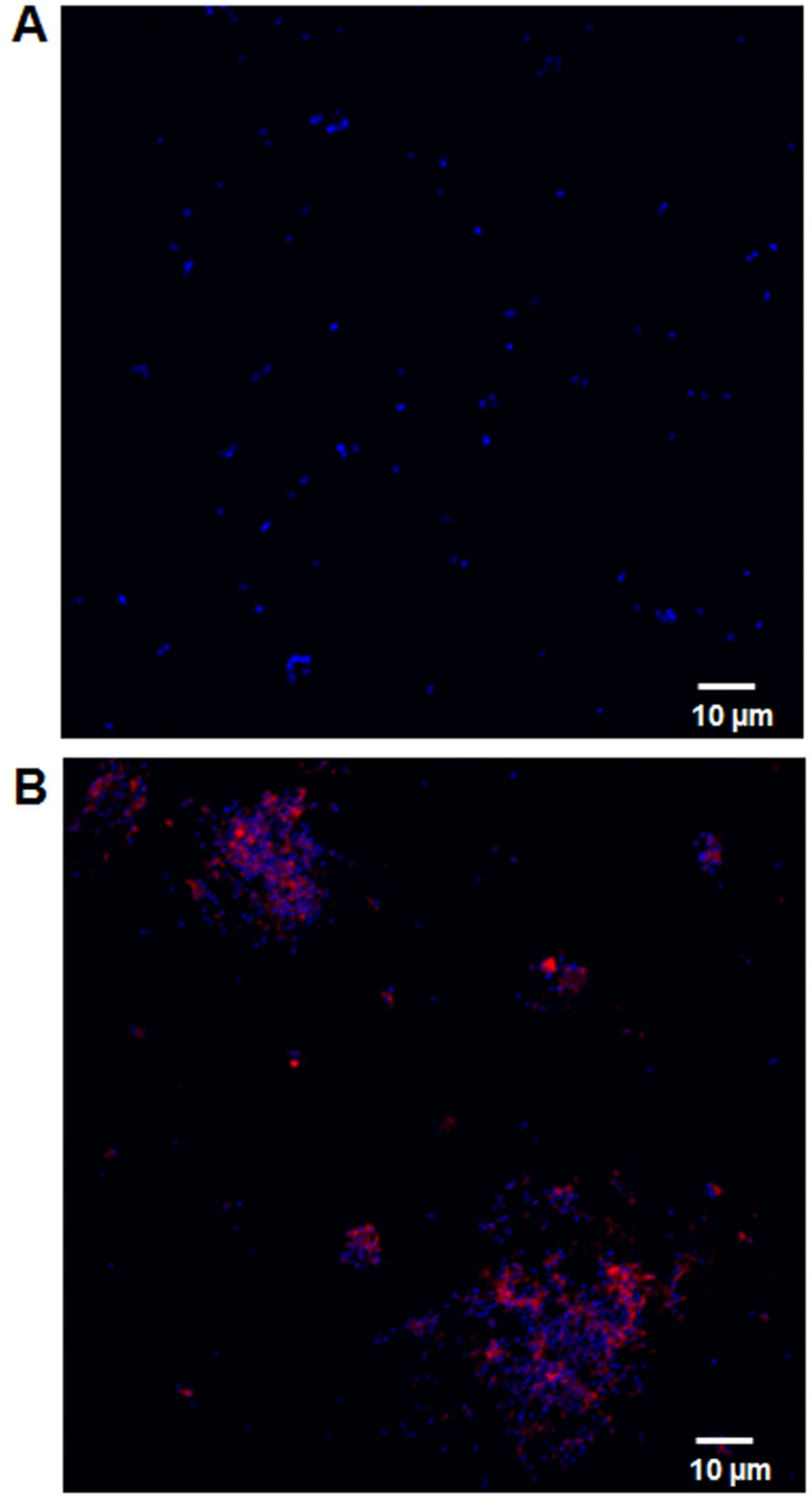
NK2A associates with *H*. *somni* cell membranes. *H*. *somni* was incubated with unconjugated Cy5 (A) or 20 μM NK2A-Cy5 conjugate for 30 min and bacterial nuclear contents were stained with DAPI. NK2A-Cy5 conjugate is shown in red and DAPI is shown in blue.

It has been previously reported that most antimicrobial proteins (AMPs) interact with lipid bilayer, however some AMPs can interact with other intracellular targets such as nucleic acids [6]. For example, Buforin II, an AMP isolated from the stomach tissue of Asian toad *Bufo bufo garagrizans*, penetrates the bacterial membranes (without damaging the cell membrane) and enters the cytosol, then binds with bacterial DNA and RNA [22]. Both our confocal microscopic and flow cytometry analysis (Figs 2 and 3) confirmed that bovine NK-lysins caused damage to *H*. *somni* cell membranes, but we wished to further evaluate whether NK-lysin peptides can also interact with other bacterial targets, specifically with *H*. *somni* DNA. Therefore, *H*. *somni* was incubated with NK2A-Cy5 conjugate (for 30 min) and observed the bacteria under confocal microscope after staining bacterial DNA with DAPI. Unconjugated or free Cy5 dye did not bind with bacteria but their nuclear materials were clearly stained with DAPI (Fig 4A). Although NK2A-Cy5 staining was visible with *H*. *somni*, we did not observe any co-localization of NK2A-Cy5 conjugate with DAPI (Fig 4B) suggesting that NK2A did not bind with DNA, but interacted with bacterial cell membrane.

**Fig 5:**
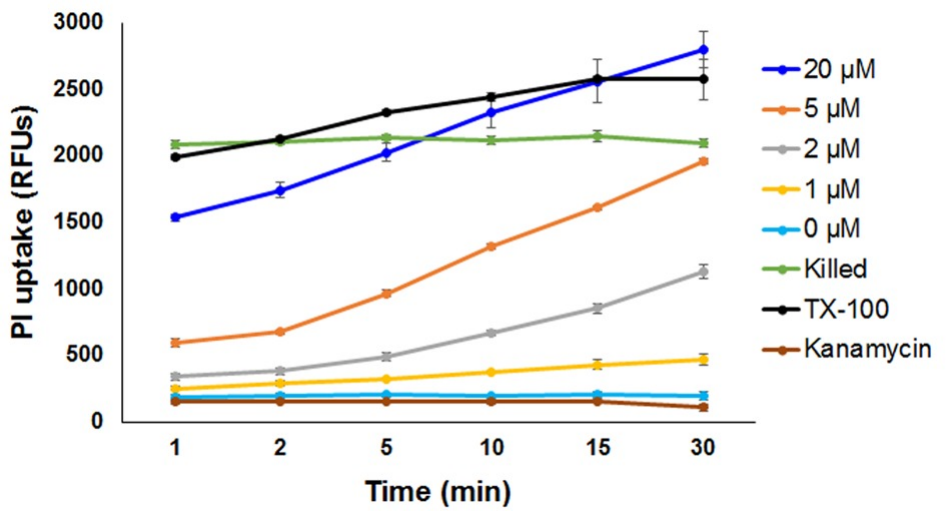
Analysis of propidium iodide uptake of *H*. *somni* following incubation with bovine NK2A peptide. *H*. *somni* was incubated with propidium iodide (PI) for 5 min followed by addition of different concentrations of NK2A peptide (1, 2, 5 and 20 μM final concentrations) and PI fluorescence was immediately read using a fluorescent microplate reader. Ethanol-killed and 1% (v/v) Triton X 100 treated *H*. *somni* were used as positive controls and untreated and kanamycin (50 μg/ml) treated *H*. *somni* were used as negative controls. Error bars indicate standard deviations of the means.

In order to examine the kinetics of NK-lysin-induced *H*. *somni* cell membrane damage, we performed propidium iodide (PI) uptake assay in a time-dependent manner following incubation of bacteria with different concentrations of NK2A. As early as 1 min post-incubation, higher PI fluorescence emission was observed with higher concentrations of NK2A (5 and 20 μM) and PI uptake continuously increased during the 30 min incubation period (Fig 5). Lower, but time-dependent increases in PI uptake was also observed with lower concentrations of NK2A (1 and 2 μM). As expected, higher PI uptake was observed with ethanol-killed and Trion X-100 treated samples while low-to-no PI uptake was observed with NK2A untreated and kanamycin treated samples (Fig 5).

**Fig 6:**
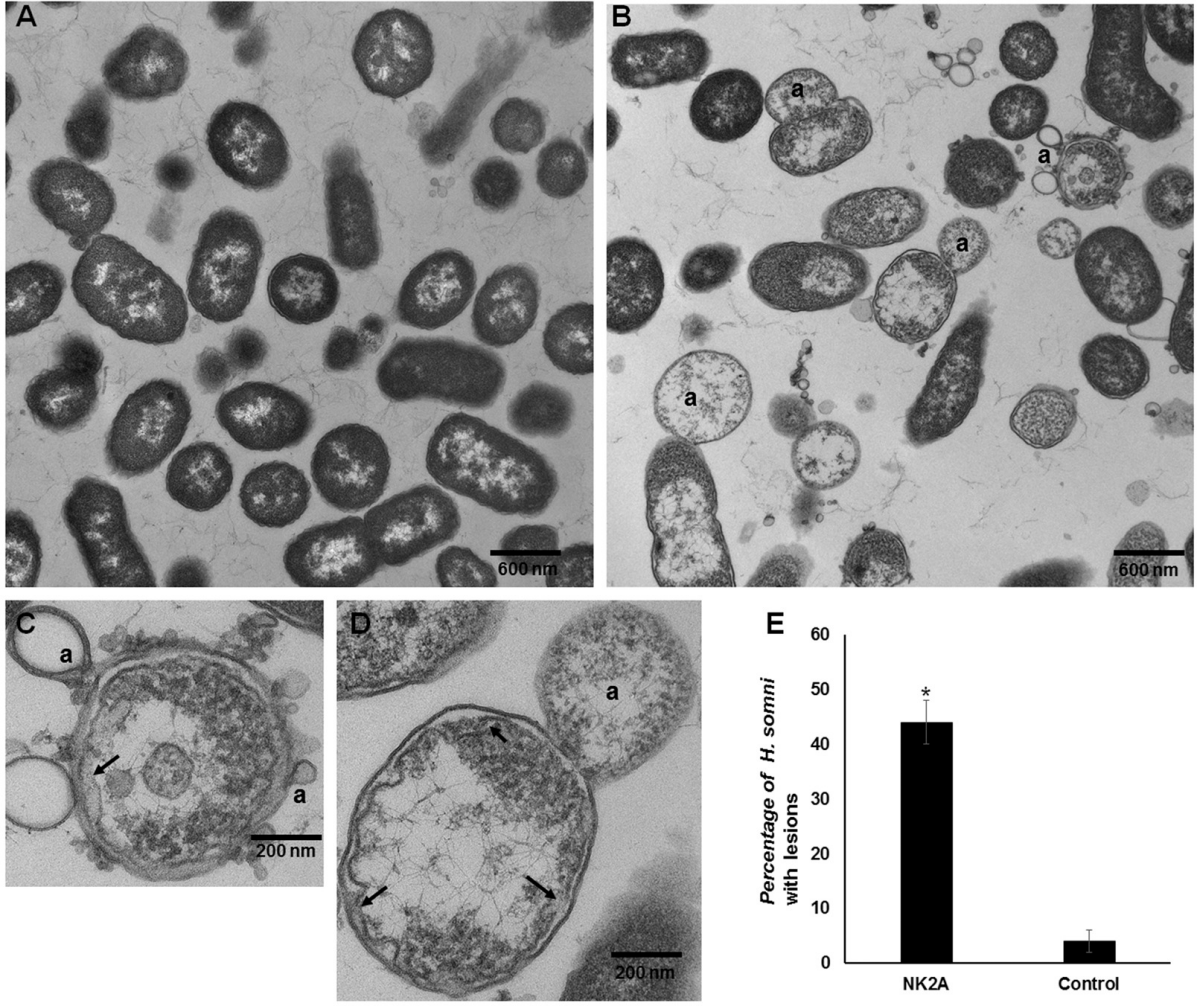
NK2A-induced damage to *H*. *somni* cell membranes. (A) Control *H*. *somni*. (B), (C), and (D) *H*. *somni* were incubated with 30 μM NK2A peptide at 37°C for 60 min. Arrows indicate damaged inner membranes and letter “a” indicates protrusions on outer membrane. (E) Percentage of *H*. *somni* with membrane damage. Means and standard deviations were calculated from six electron micrographic images of control and NK2A treated samples. (*P*<0.001)

To visualize ultrastructural damage to cell membranes, we prepared control and NK2A treated *H*. *somni* samples for transmission electron microscopic assessment. Both inner and outer membranes of control *H*. *somni* were intact and their cytosol was filled with electron dense material suggesting these bacteria are alive and healthy (Fig 6A). In contrast, NK2A treatment led to extensively damaged *H*. *somni* cell membranes and their cytosol appeared to be clear indicating cytosolic contents leakage (Fig 6B). Although the damage to the bacterial inner membranes was very clear (arrows), *H*. *somni* were also found to contain single to multiple protrusions (letter “a”) of the outer membranes (Fig 6B, 6C and 6D). Using six electron micrographic images (taken at 11,000 magnification), we calculated percent viable and dead *H*. *somni* in both control and NK2A treated samples (Fig 6E) and statistically analyzed the data using student’s *t*-test. Compared to control samples (~5% dead bacteria), significantly higher dead *H*. *somni* (~45% dead bacteria with membrane damage, protrusions and clear zones of cytosol) in NK2A treated samples, further confirming the antimicrobial activity of NK2A.

## Discussion

Both *H*. *haemolytica* and *P*. *multocida* belong to the *Pasteurellaceae* family and share many common properties, yet their relative sensitivity to NK-lysin peptides were different [14]. It has been previously reported that unlike *M*. *haemolytica* isolates, *P*. *multocida* isolates were less susceptible to antimicrobial activity of all four peptides [14]. Although *P*. *multocida* isolates showed moderate sensitive to antimicrobial activity of NK1 and NK2A peptides, *M*. *haemolytica* isolates showed enhanced sensitive to NK2A and NK2C peptides [14]. Similar to *M*. *haemolytica* isolates [14], NK2A and NK2C peptides consistently displayed the highest antimicrobial activity against *H*. *somni* isolates. Ten micromole concentrations of NK2A and NK2C peptides were able to completely eliminate viability of all *H*. *somni* isolates within 60 min incubation period. However, unlike with *P*. *multocida* and *M*. *haemolytica* isolates [14], NK1 and NK2B peptides also displayed substantial antibacterial activity against all four *H*. *somni* isolates, but at relatively higher peptide concentrations.

Amino acid sequence analysis revealed that NK2A, NK2B and NK2C (146 residues) showed higher identity to each other in comparison to NK1 (142 resides). However, all four 30-mer long synthetic NK-lysin peptides corresponding to the functional region of helices 2 and 3 showed similar hydrophobicity (40–43%) and basic residues (20–30%) such as arginine, lysine, and histidine. All four peptides also contain two conserved cysteine residues at 10^th^ (helix 2) and 20^th^ (helix 3) position. It has been previously reported that secondary structure of NK2A, NK2B and NK2C peptides were very similar while NK1 peptide differed structurally with a lower degree of helicity [14]. Despite these differences, NK1 peptide showed the highest antimicrobial activity against *E*. *coli* and *S*. *aureus* [10] while NK2A and NK2C peptides showed the highest antimicrobial activity against *M*. *haemolytica* [14] and *H*. *somni* isolates (this study). Given the similarities in amino acid residues and secondary (helical) structures among bovine NK-lysin peptides, differences in antibacterial activity among bovine NK-lysin peptides on additional bacterial pathogens should be examined in future studies.

Antimicrobial peptides (AMPs) are a diverse group of molecules which play an important role in the host innate immune response. AMPs such as granulysin and NK-lysin are well-known to kill both Gram-positive and–negative bacteria, parasites and fungi [9, 12]. Propidium iodide is widely used in flow cytometry and fluorescent microscopy to differentiate live from dead cells as it cannot cross the intact membrane of live cells (to stain DNA). In our propidium iodide uptake assay, both NK2A untreated and kanamycin treated samples did not show any increase in PI fluorescence suggesting a lack of *H*. *somni* cell membrane damage. However, time- as well as concentration-dependent increased in PI fluorescence or PI uptake in NK2A treated samples was observed strongly suggesting NK2A-induced *H*. *somni* cell membrane damage. Furthermore, incubation of *H*. *somni* with Cy5 directly conjugated NK2A peptide confirmed a lack of association of bovine NK-lysin with bacterial DNA, but rather showed an interaction with bacterial cell membranes. Although our confocal microscopic images confirmed the extensive damage to *H*. *somni* cell membranes following incubation with NK-lysin peptides as treated bacteria were positive for propidium iodide staining, the resolution of the confocal images were not adequate to identify ultrastructural changes to the bacterial cell membranes. Therefore, we assessed ultrastructural morphological changes of control and NK-lysin treated *H*. *somni* by transmission electron microscopy. As has been observed with other bacterial pathogens [10, 14], electron micrographs confirmed the rupture of *H*. *somni* inner membranes when incubated with NK2A. It has been previously reported that NK-lysin induced damage to *P*. *multocida* cell membranes with cytosolic content leakage, but without membrane-enclosed bodies [14]. Such membrane-free coagulated content leakage was not very visible with *H*. *somni* upon incubation with NK-lysin. Rather most of the cytosolic contents were observed to be attached to the bacterial outer membrane or released as outer membrane enclosed vesicles/bodies. Similar single to multiple outer membrane protrusions development have been previously reported with *Mycobacterium tuberculosis* when incubated with human granulysin [12].

Although there are many *H*. *somni* vaccines (bacterins) currently available in the market, they show limited efficacy due a variety of reasons, such as, antigenicity of vaccine strains may be different from *H*. *somni* isolates currently circulating in the field. A subunit vaccine comprised of a recombinantly expressed DR2 domain of ibpA [23] or multiple antigens expressed using a genome-based reverse vaccinology technique with a field *H*. *somni* isolate [24] showed enhanced protection in cattle against subsequent challenge with *H*. *somni*. In this study, we showed that all four NK-lysin peptides are highly effective against four different *H*. *somni* isolates, but at 10 μM or higher concentrations. Therefore, combination of NK-lysin peptides, particularly NK2A, with *H*. *somni* recombinant or subunit vaccines might provide enhanced protection in cattle against *H*. *somni* infections. However, one must consider the cost associated with AMP production particularly for large animal trials, as well as the possibility of development of resistance to AMPs by microorganisms. However, such resistance to AMPs among microbes has been shown to be very limited. Furthermore, although several AMPs are in clinical trials, the FDA has yet to approve any AMPs for medical usage. Therefore, it will be interesting to determine whether bovine NK-lysin in general, and NK2A in particular, can be used as a treatment or preventive to reduce the occurrence of BRDC in calves.

## Conclusions

Using several techniques such as bacterial killing assay, Live/Dead bacterial staining, followed by confocal laser-scanning microscopic and flow cytometry analyses have revealed that bovine NK-lysin-derived peptides are bactericidal to *H*. *somni*. Moreover, electron microscopic analyses revealed that NK-lysin-derived peptide (NK2A) caused extensive damage to *H*. *somni* inner and outer membrane, cytosolic content leakage, as well as protrusions of outer membranes. Based on our results and those observed previously [14], now we can conclude that bovine NK-lysin-derived peptide NK2A is effective against the bacteria agents involved in BRDC such as *M*. *haemolytica*, *P*. *multocida*, and *H*. *somni*.
